# Differential Expression of *MITF*, *WNT3A*, *SLC7A11*, and *EDN3* in the Shoulder ‘Bider Marking’ of Dun Mongolian Horses

**DOI:** 10.3390/ani16060967

**Published:** 2026-03-19

**Authors:** Tana An, Manglai Dugarjaviin

**Affiliations:** Inner Mongolia Key Laboratory of Equine Science Research and Technology Innovation, Inner Mongolia Agricultural University, Hohhot 010018, China; 15848154479@163.com

**Keywords:** dun, Mongolian horse, Bider markings, pigmentation, gene expression

## Abstract

(1) Background: The “Bider marking” on the shoulder of the Dun Mongolian horse represents a unique pigmentation pattern, the molecular formation mechanism of which remains incompletely understood. This study investigates the differential expression and protein localization of pigment-related genes—specifically the core transcription factor *MITF*, as well as *EDN3*, *SLC7A11*, and *WNT3A*—in the skin. The analysis focuses on three distinct regions: the dark-colored area of the ‘Bider marking’ shoulder (BIDC), the light-colored area of the ‘Bider marking’ shoulder (BILC), and the non-Bider-marked shoulder area (NBIS). The aim is to clarify their correlation with the formation of this distinctive pigmentation pattern. (2) Methods: Skin tissue samples from both the “Bider marking” and non-Bider-marked shoulder regions were collected (*n* ≥ 3). The mRNA expression levels were quantified using RT-qPCR, protein levels were analyzed through Western blotting, and protein localization was assessed via immunohistochemistry and immunofluorescence. (3) Results: Compared to the NBIS group, both the BIDC and BILC groups exhibited significantly elevated protein expression of MITF and WNT3A. Further immunofluorescence showed that the distribution of MITF protein exhibits regional specificity in the epidermis and hair follicles. In the BIDC region, the protein is localized specifically to the stratum corneum of the epidermis, the dermal papilla, and the outer root sheath of hair follicles. In contrast, the mRNA and protein expression levels of SLC7A11 and EDN3 did not display consistent patterns among the three groups, and no specific differences were observed in tissue localization. (4) Conclusions: The findings show that the specific pigmentation in dark “Bider marking” regions is closely linked to the upregulated protein levels and unique spatial patterns of *MITF* and *WNT3A*; *SLC7A11* and *EDN3* may not be primary regulators of this trait.

## Simple Summary

The ‘Bider marking’ on the shoulder of Dun Mongolian horses is a unique symmetrical pigmented patch with unclear formation mechanisms. This study analyzed the expression and localization of the MITF, WNT3A, SLC7A11, and EDN3 genes at both mRNA and protein levels by comparing skin tissues from the dark-colored area of the ‘Bider marking’ shoulder (BIDC), the light-colored area of the ‘Bider marking’ shoulder (BILC), and the non-Bider-marked shoulder area (NBIS). Results indicated that the protein expression of both *MITF* and *WNT3A* in the BIDC area was significantly higher than that in the NBIS area. The expression patterns of *SLC7A11* and *EDN3* are both constantly changing and complex; they do not demonstrate a clear association with pigmentation. Studies have shown that the upregulation of MITF and WNT3A at the protein level, along with their spatial distribution specificity, is closely related to the formation of the dark patches in ‘Bider markings.’ These findings provide important insights into the molecular mechanisms underlying this trait.

## 1. Introduction

Pigmentation in animal skin is a complex biological process [1], involving crucial physiological functions such as camouflage, social communication, thermoregulation and UV protection [2]. This process is primarily regulated by melanocytes [3], which synthesize eumelanin and pheomelanin; their varying deposition proportions within the skin and hair follicles create rich phenotypic diversity [4]. As a product of long-term domestication and selective breeding [5], domestic horses exhibit remarkable coat color diversity. Among these, the dun coloration represents an ancestral characteristic with significant research value [6]. The distinctive “Bider marking” [7] on the shoulders of dun Mongolian horses serves as an ideal model for studying localized specific pigmentation. This marking manifests as bilaterally symmetrical irregular black patches on the scapular region, commonly observed in Przewalski’s horses and Mongolian horses, and represents an autosomal genetic trait [8]. Investigating the formation mechanism of this unique marking not only helps reveal the genetic basis of pigmentation in *Equus* species but also holds practical significance for the conservation and utilization of Mongolian horse genetic resources.

Pigmentation is precisely regulated by a polygenic network [9]. The Microphthalmia-associated transcription factor (*MITF*) serves as the central transcription factor governing melanocyte differentiation and functional regulation [10], and serves as the master regulator of melanogenesis [11]. *MITF* is a key regulator of pigmentation, controlling the expression of melanin synthesis genes such as Tyrosinase (*TYR)* and Tyrosinase-Related Protein 1 (*TYRP*1) [12,13]. As a cellular signaling molecule [14], endothelin-3 (*EDN3*) promotes the proliferation, migration, and survival of melanocyte precursors, thereby influencing the ultimate distribution of melanocytes [15]. Solute carrier family 7 member 11 (*SLC7A11*) influences the redox balance of melanocytes by regulating intracellular cystine uptake and glutathione (GSH) synthesis [16]. High cystine levels promote eumelanin synthesis, whereas low levels favor pheomelanin production [1]. Wnt family member 3A (*WNT3A*), the initiating ligand of the Wnt/β-catenin pathway, is essential for determining melanocyte lineage during embryonic development [17]. Currently, although the functions of these genes in pigmentation are partially understood, their specific roles and expression patterns in horse skin pigmentation—particularly in the formation of the localized and specific “Bider marks” in Mongolian horses—still lack systematic investigation. Preliminary histological analysis has revealed differences in pigment deposition patterns within the “Bider marking” regions [7]; however, the molecular mechanisms underlying these morphological differences, especially the expression variations of key regulatory genes, remain unclear. The precise role and cooperative mechanism of the gene set (*MITF*, *EDN3*, *SLC7A11*, and *WNT3A*) in the development of “Bider markings” in dun Mongolian horses require further clarification. Previously, using dun Mongolian horses exhibiting “Bider markings” as a model, we applied multidisciplinary techniques to address two questions: first, analyzing T-box transcription factor 3 (*TBX3*) expression differences across various skin regions (shoulder, dorsal midline, croup) within the same individual [7]; and second, conducting a systematic comparison of hair follicle morphology, pigment distribution, and *TBX3* expression/localization between the marked (light/dark shoulder) and unmarked regions [18]. Based on the aforementioned research background and existing gaps, this study aims to systematically elucidate the role of key pigmentation-related regulatory genes in the formation of the unique pigment pattern known as the “Bider marking” in dun Mongolian horses. By comparing three distinct skin regions—the dark-shoulder area of the “Bider marking,” the adjacent light-shoulder area, and the normal shoulder skin without the marking—this research will comprehensively investigate the expression differences of *MITF*, *EDN3*, *SLC7A11*, and *WNT3A* at both transcriptional and translational levels, combined with their spatial localization in tissues. Specifically, the study will employ real-time quantitative PCR and Western blotting to quantitatively analyze mRNA and protein expression levels, respectively. Furthermore, immunohistochemistry and immunofluorescence staining will be used to visually observe the cellular and subcellular localization of these gene products in tissue sections. It is expected that this study will not only reveal the expression profiles and potential interactions of these genes in localized pigmentation but also provide empirical evidence for deciphering the molecular regulatory network underlying the formation of the “Bider marking.” This will thereby enhance the understanding of the genetic mechanisms governing coat color in Mongolian horses and offer theoretical support for the scientific conservation and utilization of this genetic resource.

## 2. Materials and Methods

### 2.1. Materials

**Figure 1:**
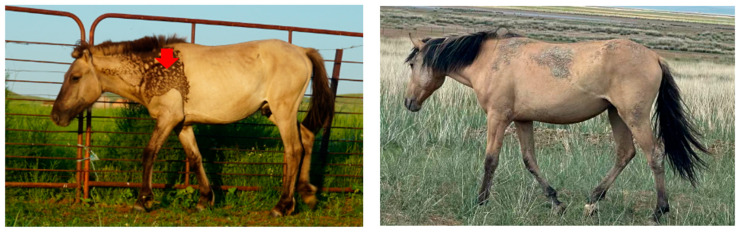
Example illustration of Dun Mongolian horses with and without Bider markings. (**Left**) dun Mongolian horse with Bider markings. (**Right**) dun Mongolian horse without Bider markings. Red arrow indicate the Bider area.

The skin tissue samples of dun Mongolian horses in this study were sourced from the Inner Mongolia Autonomous Region, China. The samples were divided into two groups based on the shoulder: horses with this marking (*n* = 3) and horses without it (*n* = 3). The marked group consisted of two female horses (aged 3 and 4 years) and one male horse (6 years old), while the unmarked group comprised three 2-year-old male horses. All horses exhibited typical dun coat phenotypes. Three 1 × 1 cm skin tissue samples were collected from different shoulder areas of each experimental horse (Figure 1): (1) the dark-colored shoulder area of the Bider marking (BIDC), (2) the light-colored shoulder area of the Bider marking (BILC), and (3) the non-Bider-marked shoulder area (NBIS). To minimize animal discomfort, sampling was carried out under combined anesthesia induced by intravenous administration of xylazine (0.01–0.02 mg/kg) and butorphanol (0.02–0.04 mg/kg) for sedation and analgesia [19].

### 2.2. Methods

This study employed methods including paraffin-embedded sectioning, RNA extraction, total protein extraction, RT-qPCR, Western blot (WB), and immunohistochemical staining, with specific experimental procedures referenced to literature [7,18]. For detailed steps, refer to Supplementary Materials Methods.

#### 2.2.1. Immunofluorescence Staining

Immunofluorescence staining was employed to accurately detect and localize the proteins—MITF (ab12039, Abcam, Cambridge, UK), WNT3A (DF6113, Affinity Biosciences, Nanjing, China), SLC7A11 (DF12509, Affinity Biosciences, Nanjing, China), and EDN3 (DF6194, Affinity Biosciences, Nanjing, China)—in skin tissue samples. The prepared sections (for detailed steps, refer to Supplementary Materials Methods) were placed in a constant temperature chamber at 65 °C and baked for 90 min to prevent the tissue samples from detaching during the experiment. The sections were dewaxed twice with xylene solution for 20 min, then hydrated and rinsed with PBS three times for 5 min each. After antigen retrieval in 95 °C boiling water for 15 min and subsequent cooling to room temperature, the tissue sections were circled with a PAP pen. Next, 100 μL of blocking buffer (1:20 goat serum) was added to the circled area, ensuring complete coverage of the sample surface, and the sections were incubated at room temperature for 2 h in a wet box. After incubation, the blocking buffer was removed, and 100 μL of primary antibody was added. The samples were then placed in a wet box and incubated overnight (16–18 h) at 4 °C. Blocking buffer was applied to the control group to distinguish between non-specific antibody binding. The following day, the samples were re-warmed for 30 min and rinsed with PBS three times for 10 min each. Subsequently, 100 μL of fluorescent secondary antibody (S0001, Affinity Biosciences, Changzhou, China) was added to the control group, and the samples were incubated for 2 h at 37 °C in a wet box shielded from light. Afterward, the secondary antibody was removed, and the samples were rinsed with PBS three times for 10 min each. Finally, DAPI was added, and the samples were incubated in a cassette for 10 min, followed by rinsing with PBS three times for 10 min each. During sealing, an anti-fluorescence quenching agent was applied in droplets to prevent fluorescence quenching, and the tissue morphology was observed under a microscope after covering.

#### 2.2.2. Statistical Analysis

All experiments in this study were performed with at least three independent biological replicates. Quantitative analysis of stained tissue sections was conducted using WCIF ImageJ 1.37c software. For each sample within a group, five non-overlapping and representative fields of view at 40× magnification were randomly selected. A consistent optical density threshold was applied to each field to identify positively stained regions. The following parameters were measured: Area, Mean Gray Value, and Integrated Optical Density (the product of area and mean gray value). The percentage of positive area (%Area) was calculated relative to the total field area. All statistical analyses were performed using GraphPad Prism 10 software. For multiple group comparisons, we first assessed whether the data met the assumptions for parametric tests (normality and homogeneity of variance). If satisfied, one-way analysis of variance (ANOVA) was employed. When ANOVA indicated a significant overall difference (*p* < 0.05), post hoc testing was conducted using Tukey’s Honestly Significant Difference (HSD) test for comprehensive pairwise comparisons, which is suitable for groups with equal sample sizes (*n* = 3) and controls the family-wise error rate. For pre-planned, specific pairwise comparisons, unpaired two-tailed *t*-tests with Bonferroni correction were applied. Significance levels are denoted as follows: * *p* < 0.05, ** *p* < 0.01, and ns (not significant) for *p* > 0.05.

## 3. Results

### 3.1. Expression and Localization of the MITF

**Figure 2:**
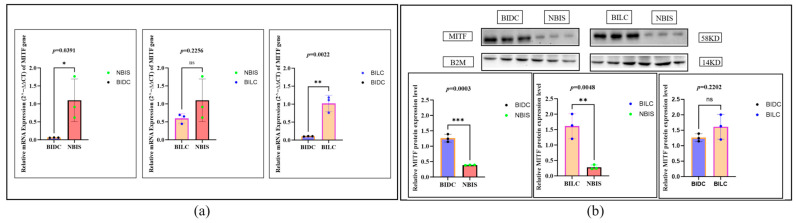
(**a**) Relative expression levels of the *MITF* gene in skin tissues of Bider and non-Bider horses from different sampling sites. (**b**) Expression levels of MITF protein in skin tissues of Bider and non-Bider horses from different sampling sites. Data are presented as mean ± SEM (*n* = 3 per group). We used Tukey’s HSD test (or Bonferroni correction). *p* < 0.05 (*), *p* < 0.01 (**/***), and *p* > 0.05 (ns). Scatter points represent individual samples.

At the mRNA level, compared with the NBIS group, *MITF* expression in the BIDC group was lower than in the NBIS group (*p* = 0.0391), with its expression reduced to about 0.07 times that of the NBIS group; there was no significant difference between BILC and NBIS (*p* = 0.2256). However, compared with the BIDC group, *MITF* mRNA expression in the BILC group was significantly higher (*p* = 0.0022), about 9.1 times that of the BIDC group (i.e., the BIDC group was downregulated to about 0.11 times the BILC group), indicating differences in transcriptional regulation among different coat color regions (Figure 2a). In contrast, at the protein level, the situation was different: compared with the NBIS group, MITF protein expression in both the BIDC and BILC groups was significantly increased (BIDC vs. NBIS, *p* = 0.0003, upregulated by about 3.3 times; BILC vs. NBIS, *p* = 0.0048, upregulated by about 6.2 times). Although the average protein expression in the BILC group was higher than in the BIDC group, there was no statistically significant difference between the two groups (*p* = 0.2202) (Figure 2b).

**Figure 3:**
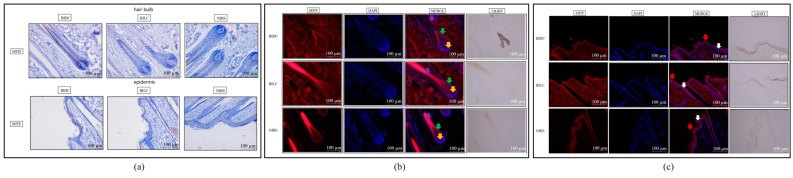
(**a**) Representative immunohistochemical staining of MITF protein in skin tissues of Bider and non-Bider horses from different sampling sites, showing its expression in the hair follicle bulb region and epidermis. (**b**) Immunofluorescence staining of MITF protein in the hair follicle bulb region. Yellow arrows indicate the dermal papilla; green arrows indicate the outer root sheath; purple arrows indicate the hair root. (**c**) Immunofluorescence staining of MITF protein in the epidermal region. Red arrows indicate the stratum corneum; white arrows indicate the basal layer. Scale bar: 100 μm.

Immunohistochemical staining indicated no significant overall specific localization or differential deposition of MITF protein among the three groups in growing hair follicles and epidermal tissues (Figure 3a and Figure S2). However, quantitative analysis revealed statistically significant differences in MITF protein expression within the hair bulb region of hair follicles between the BIDC/BILC and BIDC/NBIS comparison groups (*p* < 0.05) (Figure S1). In contrast, immunofluorescence staining unveiled distinct spatial distribution patterns: in the epidermis, MITF signal was concentrated in the stratum corneum in the BIDC group, distributed in both the stratum corneum and basal layer in the BILC group, and primarily localized to the basal layer in the NBIS group. Within hair follicles, MITF was detected in the dermal papilla and outer root sheath in the BIDC group, whereas in both the BILC and NBIS groups, it was predominantly expressed in the outer root sheath and hair bulb (Figure 3b,c and Figures S3 and S4).

### 3.2. Expression and Localization of the WNT3A

**Figure 4:**
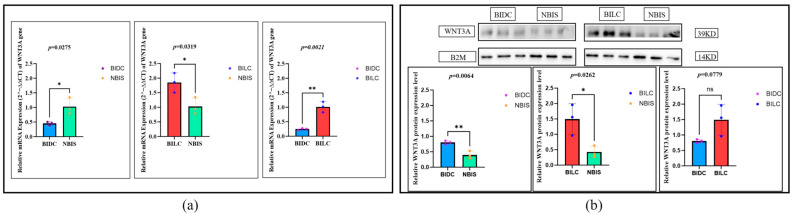
(**a**) Relative expression levels of the *WNT3A* gene in skin tissues of Bider and non-Bider horses from different sampling sites. (**b**) Expression levels of WNT3A protein in skin tissues of Bider and non-Bider horses from different sampling sites. Data are presented as mean ± SEM (*n* = 3 per group). We used Tukey’s HSD test (or Bonferroni correction). *p* < 0.05 (*), *p* < 0.01 (**), and *p* > 0.05 (ns). Scatter points represent individual samples.

At the transcriptional level, compared with the NBIS group, *WNT3A* mRNA expression was significantly downregulated in the BIDC group (*p* = 0.0275), with its expression approximately 0.5 times that of the NBIS group; in contrast, expression in the BILC group was significantly upregulated (*p* = 0.0319), approximately 1.7 times that of the NBIS group. Further comparison showed that the mRNA expression level in the BILC group was significantly higher than that in the BIDC group (*p* = 0.0021), about 3.3 times that of the BIDC group (i.e., the BIDC group’s expression was downregulated to approximately 0.3 times that of the BILC group) (Figure 4a). In contrast, at the protein level, WNT3A protein expression was significantly upregulated in both the BIDC and BILC groups compared with the NBIS group (BIDC vs. NBIS, *p* = 0.0064, upregulated about 2.1 times; BILC vs. NBIS, *p* = 0.0262, upregulated about 3.5 times), but there was no statistical difference in protein expression between the BIDC and BILC groups (Figure 4b). Overall, WNT3A showed a consistent upregulation pattern at both the transcriptional and translational levels in the BILC group, while in the BIDC group, it exhibited a discrepant pattern of mRNA downregulation but protein upregulation.

**Figure 5:**
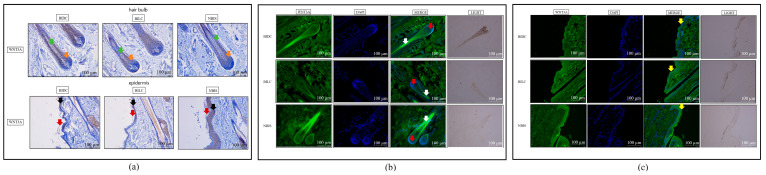
(**a**) Representative immunohistochemical staining of WNT3A protein in skin tissues of Bider and non-Bider horses. WNT3A expression is shown in the hair follicle bulb and the epidermis. Orange and green arrows indicate WNT3A localization in the hair follicle bulb and root, respectively. Red and black arrows indicate WNT3A localization in the epidermal basal layer and stratum corneum, respectively. (**b**) Immunofluorescence staining of WNT3A protein in the hair follicle bulb region. Red arrows indicate localization in the dermal papilla; white arrows indicate localization in the hair roots. (**c**) Immunofluorescence staining of WNT3A protein in the epidermal region. Yellow arrows indicate localization in the stratum corneum. Scale bar: 100 μm. *n* = 3 per group.

Immunohistochemical staining localized WNT3A protein to the epidermal basal layer, stratum corneum, and hair follicle tissues in all groups, with particularly intense deposition observed in the NBIS basal layer (Figure 5a and Figure S6). Significant differences in protein deposition within the hair bulb of growing follicles were also quantified (BIDC vs. NBIS; *p* < 0.05) (Figure S5). These localization findings were validated by immunofluorescence, which detailed WNT3A distribution in the dermal papilla, hair root, and stratum corneum, and identified a statistically significant difference in epidermal staining between BIDC and BILC (*p* < 0.05) (Figure 5b,c and Figures S7 and S8).

### 3.3. Expression and Localization of the SLC7A11

**Figure 6:**
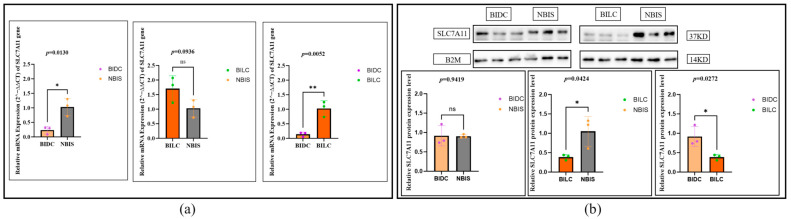
(**a**) Relative expression levels of the *SLC7A11* gene in skin tissues of Bider and non-Bider horses from different sampling sites. (**b**) Expression levels of SLC7A11 protein in skin tissues of Bider and non-Bider horses from different sampling sites. Data are presented as mean ± SEM (*n* = 3 per group). We used Tukey’s HSD test (or Bonferroni correction). *p* < 0.05 (*), *p* < 0.01 (**), *p* > 0.05 (ns). Scatter points represent individual samples.

The expression of *SLC7A11* exhibits complex multi-level regulation at both the transcriptional and translational levels. qPCR analysis showed that at the mRNA level, the expression in the BIDC group was significantly lower than in the NBIS group (*p* = 0.0130), about 0.2 times that of the NBIS group; although the BILC group was higher than the NBIS group, the difference did not reach statistical significance (*p* = 0.0936, approximately 1.6 times that of the NBIS group). However, the mRNA expression level in the BILC group was significantly higher than that in the BIDC group (*p* = 0.0052), about 5 times that of the latter (i.e., the BIDC group expression was downregulated to approximately 0.2 times that of the BILC group) (Figure 6a). In sharp contrast, the protein expression pattern showed that the protein levels of the BIDC group were comparable to those of the NBIS group, with no significant difference (*p* = 0.9419, about 0.9 times that of the NBIS group); whereas the protein expression in the BILC group was significantly lower than that in the NBIS group (*p* = 0.0424, down to about 0.4 times) and the BIDC group (*p* = 0.0272, the protein expression in the BIDC group was about 2.2 times that of the BILC group). This significant divergence between mRNA and protein expression trends suggests that SLC7A11 may be subject to active post-transcriptional regulation (Figure 6b).

**Figure 7:**
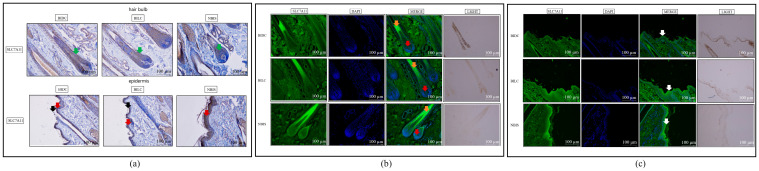
(**a**) Representative immunohistochemical staining of SLC7A11 protein in skin tissues of Bider and non-Bider horses, showing its expression in the hair follicle bulb and epidermis. Green arrows indicate SLC7A11 in hair follicles. Red and black arrows indicate SLC7A11 localization in the epidermal basal layer and stratum corneum, respectively. (**b**) Immunofluorescence staining of SLC7A11 protein in the hair follicle bulb region. Red arrows indicate localization in the dermal papilla; orange arrows indicate localization in the hair roots. (**c**) Immunofluorescence staining of SLC7A11 protein in the epidermal region. White arrows indicate localization in the stratum corneum. Scale bar: 100 μm. *n* = 3 per group.

Further immunohistochemical histology analysis revealed widespread positive localization of the SLC7A11 protein in the epidermal basal layer, stratum corneum, and hair follicle structures across all three groups (Figure 7a). Semi-quantitative assessment indicated statistically significant differences in protein deposition levels within both anagen hair follicles and epidermal tissues. Specifically, in the hair bulb region of the follicles, a significant difference was observed for the BILC/NBIS comparison (*p* < 0.01) (Figure S9). Within the epidermal layer, significant differences were found for both the BILC/NBIS and BIDC/NBIS comparisons (*p* < 0.01 for each) (Figure S10). This broad tissue distribution was corroborated by immunofluorescence staining, which confirmed the extensive presence of SLC7A11 in the epidermal basal layer, stratum corneum, and hair follicles (Figure 7b,c and Figures S11 and S12).

### 3.4. Expression and Localization of the EDN3

**Figure 8:**
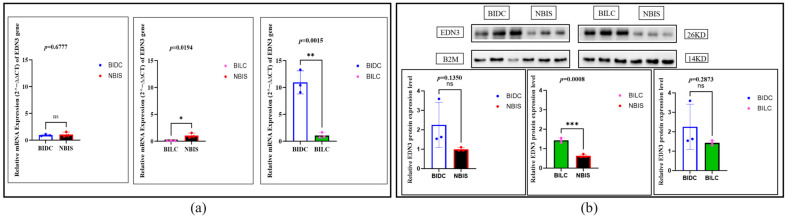
(**a**) Relative expression levels of the *EDN3* gene in skin tissues of Bider and non-Bider horses from different sampling sites. (**b**) Expression levels of EDN3 protein in skin tissues of Bider and non-Bider horses from different sampling sites. Data are presented as mean ± SEM (*n* = 3 per group). We used Tukey’s HSD test (or Bonferroni correction), with *p* < 0.05 (*), *p* < 0.01 (**/***) and *p* > 0.05 (ns). Scatter points represent individual samples.

*EDN3* exhibits a multi-layered, complex regulatory pattern. Transcript analysis shows that at the mRNA level, there is no significant difference in expression between the BIDC group and the NBIS group (*p* = 0.6777, approximately 1.0 times that of the NBIS group), but the expression in the NBIS group is significantly higher than in the BILC group (*p* = 0.0194, the BILC group downregulated to about 0.1 times that of the NBIS group). Notably, the mRNA expression in the BIDC group is significantly upregulated compared to the BILC group (*p* = 0.0015), approximately 10.6 times that of the BILC group (Figure 8a). At the protein level, the expression pattern differs: there is no significant difference in protein expression between the BIDC group and the NBIS group (*p* = 0.1350, BIDC group is about 1.8 times that of NBIS group), whereas the protein expression in the BILC group is significantly higher than in the NBIS group (*p* = 0.0008, upregulated by about 2.2 times); there is no statistical difference in protein expression between the BIDC group and the BILC group (*p* = 0.2873, BIDC group is about 1.1 times that of the BILC group). This reveals that EDN3 exhibits inconsistent regulation at the transcriptional and translational levels (Figure 8b).

**Figure 9:**
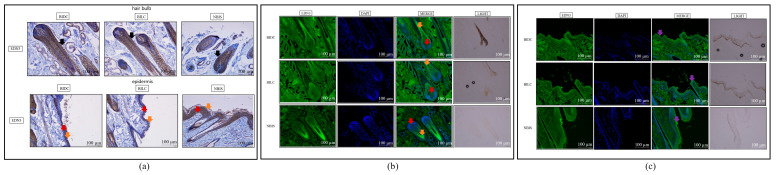
(**a**) Representative immunohistochemical staining of EDN3 protein in skin tissues of Bider and non-Bider horses, showing its expression in the hair follicle bulb and epidermis. Black arrows indicate EDN3 in hair follicles. Red and orange arrows indicate EDN3 localization in the epidermal basal layer and stratum corneum, respectively. (**b**) Immunofluorescence staining of EDN3 protein in the hair follicle bulb region. Red arrows indicate localization in the dermal papilla; orange arrows indicate localization in the hair roots. (**c**) Immunofluorescence staining of EDN3 protein in the epidermal region. Purple arrows indicate localization in the stratum corneum. Scale bar: 100 μm. *n* = 3 per group.

Immunohistochemistry demonstrated widespread EDN3 protein localization in the epidermal basal layer, stratum corneum, and hair follicle structures across all groups (Figure 9a and Figures S13 and S14), a finding corroborated by immunofluorescence staining (Figure 9b,c and Figure S16). Semi-quantitative analysis further identified significant differences in EDN3 deposition within anagen hair follicle tissues between the BIDC/NBIS and BILC/NBIS comparison groups (*p* < 0.05) (Figure S15).

## 4. Discussion

This study examines the expression and localization of *MITF*, *WNT3A*, *SLC7A11*, and *EDN3* in the skin tissues of dun Mongolian horses from ‘Bider marking’ the dark-colored shoulder area (BIDC), light-colored shoulder area (BILC), and non-Bider-marked shoulder area (NBIS), offering new insights into this distinct pigmentation pattern.

The key biological insight is that the formation of the ‘Bider marking’ is governed by a coordinated regulatory network rather than linear control by a single gene. Specifically, MITF and WNT3A proteins are consistently upregulated and exhibit region-specific localization—particularly of MITF in the epidermis and hair follicles—which closely correlates with pigmentation in the dark regions. In contrast, *SLC7A11* and *EDN3* display more complex expression profiles, suggesting they may play indirect or modulatory roles. Together, these findings highlight that precise spatiotemporal regulation of both transcription factor levels and upstream signaling pathways is essential for the development of this phenotypic trait. *MITF* is a core regulatory factor governing melanocyte development, differentiation, and pigmentation [18,19,20]. In the context of the “Bider marking” in Dun Mongolian horses, our study revealed a complex regulatory pattern for *MITF*. While its protein expression was significantly elevated in both the BIDC and BILC regions compared to NBIS, aligning with its established role as a positive regulator of melanogenesis, a notable paradox was observed specifically in the BIDC region: downregulated mRNA expression alongside upregulated protein levels.

This discrepancy between transcriptional and translational outputs primarily highlights a potential layer of post-transcriptional or translational regulation specific to the intensely pigmented BIDC area. We hypothesize that several mechanisms, supported by broader literature on MITF and protein regulation, could underlie this observation: (1) enhanced stability or reduced degradation of the MITF protein in the BIDC microenvironment [21]; (2) increased translational efficiency of *MITF* mRNA mediated by RNA-binding proteins or signaling pathways [22]; or (3) feedback regulation where MITF protein inhibits its own transcription [23]. These remain testable hypotheses for future investigation, as our current experimental design does not include direct mechanistic validation. Future work employing protein stability assays (e.g., cycloheximide chase) and translational efficiency analyses (e.g., polysome profiling) would be crucial to distinguish between these possibilities.

The critical role of *MITF* in spatial pigment pattern formation is conserved, as seen in the inhibition by ALX3 directing stripe formation in African striped mice [24]. In horses, *MITF* mutations are linked to white spotting patterns [25]. Beyond expression levels, our immunofluorescence results revealed region-specific differences in MITF protein localization, which may offer functional clues. The intense *MITF* signal in the stratum corneum of the BIDC region could suggest more active processing or retention of melanin in keratinocytes. Furthermore, its distinct localization within the dermal papilla and outer root sheath of hair follicles might be associated with the maintenance or differential activity of melanocyte stem cells, potentially contributing to the stability of the marking. In summary, our data uncover a decoupling of *MITF* mRNA and protein regulation in the BIDC region, presenting a new facet of its role in localized pigmentation. Elucidating the precise molecular mechanisms behind this discrepancy represents a key direction for future research into the establishment of the “Bider marking.”

The Wnt/β-catenin pathway, initiated by key ligands such as *WNT3A*, plays a fundamental role in melanocyte biology by promoting the expression of *MITF* and subsequently regulating melanin synthesis [26]. This study demonstrates that WNT3A protein is highly expressed in the marked regions (BIDC, BILC), with its expression pattern consistent with that of MITF protein. The obtained data provides evidence supporting the active role of the Wnt/MITF pathway in driving the pigmentation of ‘Bider marking’. Research on *WNT3A* indicates that abnormalities in the Wnt signaling pathway can lead to defects in pigment cell distribution [27]. In this study, the upregulation of *WNT3A* signaling may directly lead to the nuclear translocation of β-catenin, thereby activating the transcriptional expression of *MITF* and ultimately driving the expression of downstream melanin synthesis genes such as *TYR*, which promotes the production of eumelanin [28]. The widespread localization of *WNT3A* in hair follicles and the epidermis across different samples further demonstrates that Wnt signaling is a fundamental and crucial pathway in skin pigmentation [29]. The activation of this pathway in the unique pigment patterns of Mongolian horses underscores its conserved and vital role, which is also present in other mammals.

The roles of *SLC7A11* and *EDN3* in the formation of the ‘Bider marking’ appear to be complex and potentially indirect. *SLC7A11* influences the redox balance of melanocytes [16], where elevated cystine levels promote eumelanin (black) synthesis, while reduced levels favor pheomelanin (yellow) production [1]. In this study, the expression pattern of *SLC7A11* was inconsistent; its mRNA expression was significantly higher in the NBIS region compared to BIDC, yet no significant difference was observed in protein levels between BIDC and NBIS. This inconsistency, along with its relatively broad localization across groups, suggests that *SLC7A11* may have limited direct regulatory effects on the formation of the ‘Bider marking.’ Its role may be more focused on maintaining redox balance through glutathione synthesis, thereby ensuring the survival and function of melanocytes, rather than directly determining dark/light patterns. Findings in rex rabbits partially support this perspective, as *SLC7A11* exhibits the highest expression level in protein yellow (PY) fur, indicating its association with pheomelanin deposition, which contrasts with the eumelanin-dominated patterns observed in our horse model [30].

Similarly, *EDN3* is recognized as a significant factor in promoting the survival and proliferation of melanocytes [15]. However, neither the mRNA nor protein expression levels of EDN3 exhibited a consistent trend that correlated clearly with pigment depth across the three groups. This complex expression pattern suggests that *EDN3* may primarily act as a microenvironmental factor influencing melanocyte survival and proliferation [31], while its direct contribution to the formation of specific pigment patterns may not be predominant in this model. In summary, the roles of *SLC7A11* and *EDN3* extend beyond direct pattern determination. They appear to function as crucial components of the supporting microenvironment—*SLC7A11* modulating biochemical conditions and *EDN3* providing trophic support—that interacts with the core patterning signal (*WNT3A*) and the master transcriptional regulator (*MITF*). This integrated network perspective explains their non-linear expression patterns and underscores that the ‘Bider mark’ arises from the concerted action of positional, transcriptional, metabolic, and survival signals.

The formation of the dark ‘Bider marking’ regions is likely a result of the activation of the *WNT3A* signaling pathway, which upregulates MITF protein expression. Elevated levels of *MITF* promote eumelanin synthesis by driving the expression of downstream genes, such as *TYR*. The specific spatial distribution of these key proteins, particularly the enrichment of *MITF* in the epidermal stratum corneum of BIDC regions, provides a finely tuned regulatory mechanism that precisely controls pigment deposition, ultimately resulting in the characteristic symmetrical shoulder patch pattern. The discrepancy between mRNA and protein levels of *MITF* in BIDC regions suggests the presence of unelucidated post-transcriptional control mechanisms that maintain high *MITF* activity in specific areas. The complex expression patterns of *SLC7A11* and *EDN3* indicate that their roles are more auxiliary, likely primarily involved in maintaining melanocyte health and metabolic states rather than directly participating in pattern formation. From an evolutionary perspective, equine coat color polymorphisms typically represent adaptations to environmental pressures, such as camouflage, thermoregulation, or sexual selection [32].

The distinctive “Bider markings” of Mongolian horses may represent an adaptation to their local environment, with the genes identified in this study (*MITF*, *WNT3A*, *EDN3*, *SLC7A11*) forming part of the complex genetic network underlying this adaptation. The genetic mechanisms of pigmentation are highly conserved among vertebrates [33]. Mutations in the *MITF* gene are well-established causes of human pigmentary disorders [34], which are characterized by pigmentary abnormalities and sensorineural hearing loss [35]. This highlights the gene’s crucial role in dun neural crest-derived cells [36]. The essential role of MITF in the neural-crest-to-melanocyte development pathway has been further underscored by evidence from model organisms [37]. This conservation makes the Mongolian horse a valuable model for understanding how this core network is regulated to produce specific localized pigment patterns.

This study systematically reveals, for the first time at both mRNA and protein levels, the specific high-expression patterns of *MITF*, *EDN3*, and *SLC7A11* in the “Bider marking” region of Mongolian horses, correlating the expression of these genes with specific pigmentation phenotypes. Innovatively, it integrates and analyzes multiple functionally related pigment genes (*MITF*, *EDN3*, *SLC7A11*, *WNT3A*) within the same localized pigmentation model, providing insights into their synergistic effects. The findings not only contribute to elucidating the molecular mechanisms underlying horse coat color formation but also offer valuable insights into the universal yet complex biological phenomenon of localized pigmentation in animals.

A limitation of this study is its relatively small sample size, which may constrain the generalizability of the findings; it primarily focuses on gene expression levels without functional experimental validation; the number of genes investigated is limited; and upstream regulatory mechanisms, such as epigenetic regulation, have not been thoroughly explored. Future research could enhance the statistical power of the conclusions by increasing the sample size, validating gene functions through genetic manipulation experiments, systematically uncovering relevant gene networks using high-throughput approaches like transcriptome sequencing, and further investigating upstream regulatory mechanisms to provide a more comprehensive understanding of the formation of the “Bider” marking.

## 5. Conclusions

This study analyzed the skin tissues of Dun Mongolian horses with Bider markings in the dark-colored shoulder area (BIDC), light-colored shoulder area (BILC), and non-Bider-marked shoulder area (NBIS) and found that the pigment deposition unique to the dark Bider-marked areas is closely associated with the significant increase in MITF and WNT3A protein levels.

## Data Availability

The original contributions presented in the study are included in the article; further inquiries can be directed to the corresponding authors.

## Supplementary Materials

The following supporting information can be downloaded at: https://www.mdpi.com/article/10.3390/ani16060967/s1, Supplementary Materials Methods and Supplementary Materials Results. Supplementary Materials Methods include the following 7 titles: S1: Paraffin Embedding and Sectioning; S2: RNA Extraction, Quality Testing, and cDNA Synthesis; S3: Real-Time Fluorescence Quantitative PCR (RT-qPCR); S4: Extraction of Total Protein; S5: Western Blot Analysis; S6: Immunohistochemical Staining; S7: Statistical analysis; Table S1: Primer sequence information of target gene and reference gene; Supplementary Materials Results include the following 16 titles: Figure S1: Quantitative analysis of immunohistochemical staining intensity (MITF) in hair bulb, Figure S2: Quantitative analysis of immunohistochemical staining intensity (MITF) in epidermis, Figure S3: Quantitative analysis of immunofluorescence staining intensity (MITF) in hair bulb, Figure S4: Quantitative analysis of immunofluorescence staining intensity (MITF) in epidermis, Figure S5: Quantitative analysis of immunohistochemical staining intensity (WNT3A) in hair bulb, Figure S6: Quantitative analysis of immunohistochemical staining intensity (WNT3A) in epidermis, Figure S7: Quantitative analysis of immunofluorescence staining intensity (WNT3A) in hair bulb, Figure S8: Quantitative analysis of immunofluorescence staining intensity (WNT3A) in epidermis, Figure S9: Quantitative analysis of immunohistochemical staining intensity (SLC7A11) in hair bulb, Figure S10: Quantitative analysis of immunohistochemical staining intensity (SLC7A11) in epidermis, Figure S11: Quantitative analysis of immunofluorescence staining in- tensity (SLC7A11) in hair bulb, Figure S12: Quantitative analysis of immunofluorescence staining intensity (SLC7A11) in epidermis, Figure S13: Quantitative analysis of immunohistochemical staining intensity (EDN3) in hair bulb, Figure S14: Quantitative analysis of immunohistochemical staining intensity (EDN3) in epidermis, Figure S15: Quantitative analysis of immunofluorescence staining intensity (EDN3) in hair bulb, Figure S16: Quantitative analysis of immunofluorescence staining intensity (EDN3) in epidermis.

## Abbreviations

Abbreviated as follows:

BILC	Bider light-colored shoulder Dun Mongolian horses
BIDC	Bider dark-colored shoulder Dun Mongolian horses
NBIS	Non Bider shoulder Dun Mongolian horses
