# Supplementary material for: Differential Expression of MITF, WNT3A, SLC7A11, and EDN3 in the Shoulder ‘Bider Marking’ of Dun Mongolian Horses
Source: Animals (Basel). 2026 Mar 19;16(6):967. doi: 10.3390/ani16060967 (PMC13023297; doi:10.3390/ani16060967)
Supplement: Supplementary file 1 [file animals-16-00967-s001.zip › Supplementary Materials Methods.pdf]

SI Methods

S1. Paraffin Embedding and Sectioning

Fixed tissues were removed from the 4% paraformaldehyde solution (Solarbio Science & Technology Co., Ltd., Beijing, China) and sequentially dehydrated in ethanol concentrations of 75%, 85%, and 95% and twice in 100% ethanol to remove water while preserving tissue morphology. Dehydrated tissues were then treated with xylene in three 20 min cycles to achieve transparency. For wax infiltration, tissues were immersed in a soft wax–xylene mixture for 30 min, followed by full immersion in soft wax for 30–45 min under a continuous vacuum (0.05 MPa) to enhance wax penetration and eliminate air bubbles. They were then transitioned to a hard wax mixture for 20 min and deeply embedded in hard wax for up to 3 h under vacuum to ensure uniform coating and structural stability. The wax-embedded tissues were allowed to solidify for 24 h, wrapped in tinfoil to preserve shape and trimmed to expose the desired tissue region for sectioning. Using a microtome (Leica Microsystems, Wetzlar, Germany), wax blocks were sliced into ultrathin sections (5 μm thickness), unfolded on a 40 ◦C spreader to form flat sheets, and transferred to adhesion microscope slides (CITOTEST, Jiangsu, China). The slides were baked in a 65 ◦C oven for 2 h to improve adhesion and remove residual solvents, fully preparing them for subsequent staining and analysis [1].

S2. RNA Extraction, Quality Testing, and cDNA Synthesis

Tissue samples were finely ground into a powder using the magnetic bead method. To prevent RNA degradation, all procedures were performed under frozen conditions. RNA was extracted following Invitrogen's TRIzol method (Invitrogen, Carlsbad, CA, USA), yielding 30 μL of total RNA per sample. The purity and concentration of RNA were assessed using an enzyme marker, with acceptable purity indicated by a 260/280 ratio of 1.9 to 2.2 and integrity confirmed by RIN values between 6.3 and 7.4. The extracted RNA was subsequently converted into cDNA through a reverse transcription process. For the reverse transcription reaction, 2 μL of 5× PrimeScript RT Master Mix (Perfect Real Time) (TaKaRa Bio Inc., Dalian, China), which included random primers, was used. The RNA concentration was carefully adjusted to ensure the total RNA in the reaction system (10 μL) did not exceed 500 ng, optimizing reverse transcription efficiency. RNase-free dH2O was added to bring the total reaction volume to 10 μL. The reaction conditions consisted of an initial incubation at 37 ◦C for 15 min, followed by a brief hold at 85 ◦C for 5 s. The resulting cDNA was stored briefly at 4 ◦C before being transferred to a -20 ◦C freezer for long-term preservation [1].

S3. Real-Time Fluorescence Quantitative PCR (RT-qPCR)

Fluorescence quantitative PCR (RT-qPCR) technology was utilized to measure the expression levels of the MITF、WNT3A、SLC7A11、EDN3 gene, using B2M as the reference gene. Gene sequences were retrieved from NCBI, and primers were designed using Primer Premier 5.0 software. The β2-microglobulin (B2M), a housekeeping gene recognized for its stable expression across various cells and tissues, was used to compare the relative levels of MITF、WNT3A、SLC7A11、EDN3 mRNA, as described in a previous study [1-2]. The primers were synthesized by Shanghai Sangon Biotech, and primer sequences are given in Table S1. Before the formal experiment, we validated the amplification efficiency and specificity of the primers through preliminary tests. Amplification efficiency was calculated using a standard curve to ensure it fell within the ideal range

of 90–110%. Product specificity was confirmed by melting curve analysis, requiring a single sharp peak, indicating the specific amplification of the target product and effectively excluding non-specific amplifications such as primer dimers. Following the TB Green Kit protocol, each sample was amplified in triplicate to ensure experimental reliability. The 25 μL reaction system comprised 12.5 μL of TB Green™ Premix Ex Taq™ II (TaKaRa Bio Inc., Dalian, China), 1 μL each of forward and reverse primers (10 μmol/μL), 2 μL of cDNA template, and 8.5 μL of dd H2O. Thermal cycling conditions included an initial pre-denaturation step at 95 ◦C for 30 s, followed by 40 cycles of denaturation at 95 ◦C for 5 s, annealing at 60 ◦C for 33 s, and extension at 72 ◦C for 30 s, conducted on a BIO-RAD CFX96™ Optics Module (BIO-RAD, Hercules, CA, USA). Post-reaction analysis provided the amplification curve, melting curve, and Ct values. Quantitative results were calculated using the $2^{-\Delta\Delta CT}$ method and analyzed using GraphPad Prism 10 software for visualization and statistical analysis.

Before conducting the ANOVA and t-tests, we performed normality and homogeneity of variance tests on the data from target gene expression across the shoulder regions. The normality of the data was assessed using the Shapiro–Wilk test, and the results indicated that all data sets followed a normal distribution (p-values > 0.05). Additionally, Levene's test was used to assess the homogeneity of variance across the groups, revealing no significant differences in variance (p-values > 0.05), confirming that the variances were consistent across the groups [1].

Table S1 Primer sequence information of target gene and reference gene

**primer name**	**primer sequences（5'-3'）**	**usage**
B2M-F	CTCTACTTTGGCCGCTATGTC	reference gene
B2M-R	CCACTTCTAAGCTGCCAGGA	
MITF-F	TCAGCATCACGCAGACCTAACTTG	target gene
MITF-R	GCTGTAGGCTTGGTTGCTCTCG	
WNT3A-F	GCCATCGGTGACTTCCTCAA	
WNT3A-R	ACCTTGAAGTGGGTGTAGCG	
SLC7A11-F	TTTCAAGGTGCCGCTCTTCATCC	
SLC7A11-R	CAGAGTGATGACGAAGCCAATCCC	
EDN3-F	ACCTCCGCCGCAGGATTGG	
EDN3-R	GGACGCCACACTGCCATCATG	

S4. Extraction of Total Protein

Skin samples were removed from the -80 ℃ freezer and immediately placed into liquid nitrogen, where they were ground into a fine powder using a tissue homogenizer. To prevent degradation, the entire process was conducted under frozen conditions. The powdered samples were transferred to 1.5 mL centrifuge tubes, with 1 mL of lysate added for every 100 mg of tissue. Following the manufacturer's instructions, a lysis buffer (Beyotime Biotechnology Co., Ltd., Shanghai, China) and protease inhibitor (Beyotime Biotechnology Co., Ltd., Shanghai, China) were mixed at a 99:1 ratio, and 1 mL of this mixture was thoroughly combined with the tissue powder for 1 min. All lysis steps were performed on ice or at 4 ℃ to maintain protein integrity. The tubes were then incubated on ice for 1 h to ensure complete lysis, followed by centrifugation at 12,000 rpm for 15 min at 4 ℃. The resulting supernatant, containing the total protein, was carefully transferred to new centrifuge tubes and stored at −20 ℃ for future experiments. Protein concentration was

determined using the BCA Protein Quantitation Kit (Beyotime Biotechnology Co., Ltd., Shanghai, China). A BCA working solution was prepared at a 50:1 ratio, and bovine serum albumin (BSA) standard solutions of varying concentrations were prepared alongside the test protein samples. Standards and samples were dispensed into a 96-well plate, followed by the addition of BCA working solution. The plate was sealed and incubated at 37 ◦C for 30 min to ensure a complete reaction. After incubation, the plate was cooled to room temperature, and a microplate reader was used to measure absorbance at 562 nm. A standard curve was generated from three replicates of the standards, plotting concentration against absorbance. Protein concentrations of the test samples were calculated by comparing their absorbance values to the standard curve [1].

S5. Western Blot Analysis

MITF、WNT3A、SLC7A11、EDN3 protein was separated, detected, and identified using SDS-PAGE electrophoresis and antibody incubation techniques. To ensure complete denaturation of the protein samples prior to electrophoresis and maintain the accuracy of subsequent analyses, the supernatant obtained in the previous step (Section 2.6) was boiled at 99 ◦C for 10 min. An SDS-PAGE gel (Solarbio Science & Technology Co., Ltd., Beijing, China) was prepared, consisting of an 15% and 10% resolving gel and a 5% stacking gel. During the electrophoresis process, the gel concentration phase was conducted at a voltage of 80 V for 30 min, followed by a separation phase at 110 V for 60 min. This setup effectively separated proteins based on their molecular weights, resulting in distinct protein bands. Following electrophoresis, the proteins were transferred to a membrane for 55 min at a current of 350 mA. To evaluate the effectiveness of the transfer, the membranes were stained with Ponceau S solution for 5 min. Unbound dye and impurities were removed with multiple 10 min washes using PBST buffer. To minimize nonspecific binding, the membranes were blocked with a 5% skim milk solution for 2 h, followed by overnight incubation at 4 ◦C with primary antibodies. The MITF protein was detected using a MITF-specific mouse monoclonal antibody (Catalog # ab12039, dilution 1:1000, Abcam, Cambridge, UK). The WNT3A protein was labeled using a rabbit polyclonal antibody (Catalog # DF6113, dilution 1:500, Affinity Biosciences, Jiangsu, China). The SLC7A11 (xCT) protein was detected using a rabbit polyclonal xCT antibody (Catalog # DF12509, dilution 1:500, Affinity Biosciences, Jiangsu, China). The EDN3 protein was probed with a corresponding rabbit polyclonal antibody (Catalog # DF6194, dilution 1:500, Affinity Biosciences, Jiangsu, China). The endogenous control protein, β-2-microglobulin (B2M), was detected using a rabbit polyclonal antibody (Catalog # DF6458, dilution 1:500, Affinity Biosciences, Jiangsu, China). After incubation for up to 16-18 h, unbound primary antibodies were removed by washing the membranes three times for 10 min each with PBST buffer. A secondary antibody, goat anti-rabbit IgG (H + L) HRP (#S0001, 1:5000 dilution, Affinity Biosciences, Jiangsu, China) and Goat Anti-Mouse IgG (H+L) HRP (#S0002, 1:5000 dilution, Affinity Biosciences, Jiangsu, China), was added and incubated for 2 h at 37 ◦C on a shaker to facilitate binding to the primary antibody, forming a detectable complex. After incubation, the membrane was washed three times for 10 min each with PBST buffer to remove the unbound secondary antibody. Protein bands were visualized using an enhanced chemiluminescence (ECL) detection system (Thermo Fisher Scientific Inc., Waltham, MA, USA) with the following exposure times: MITF, 300 s; WNT3A, 200 s; SLC7A11, 48 s; EDN3, 240 s; and the internal control B2M, 100 s.

S6. Immunohistochemical Staining

Immunohistochemical staining was employed to detect and localize MITF、WNT3A、SLC7A11、EDN3 protein in skin tissue samples. The sections prepared in Section 1. were first baked in an oven at 65 ◦C for 2 h to ensure optimal adhesion to the slides and minimize background staining. Following baking, the sections were placed into xylene solution twice for 20 min each time to achieve clearing, then processed with a series of alcohol gradient dehydration steps to thoroughly remove the wax from the sections. In the antigen retrieval stage, sodium citrate repair solution was used to treat the sections. This treatment exposed and restored the antigenic epitopes within the tissues, enhancing the sensitivity and specificity of the immunostaining process. The immunostaining protocol was carefully followed according to the reagent instructions. For the primary antibody titration, 100 μL of MITF、WNT3A、SLC7A11、EDN3 antibody, diluted at a 1:200, was applied to the sections. Control sections were treated with the dilution buffer only, without the primary antibody, to assess background staining levels. The primary antibody was incubated overnight at 4 ◦C to allow for sufficient binding to the target antigen. The next day, the sections were removed from the refrigerator and allowed to return to room temperature. They were then rinsed three times with PBS buffer to remove any unbound antibodies and impurities. For DAB (diaminobenzidine) color development, the development time was strictly controlled at 4 min to prevent over- or under-development of the staining. Following DAB development, the sections were counterstained with hematoxylin for 3 min to highlight tissue structures. Excess stain was removed by rinsing the sections under running water, which helped enhance the clarity of the staining results. The sections were then subjected to a gradient alcohol dehydration and transparency treatment to achieve optimal clarity. Finally, the sections were sealed with neutral gum and allowed to dry naturally before being observed and photographed under a microscope for protein localization.

S7. Statistical analysis

All experimental results in this study are based on at least three independent repetitions, and statistical analysis was conducted accordingly. We conducted a quantitative analysis of the grayscale values in stained sections utilizing WCIF ImageJ 1.37c software. Subsequently, all data were statistically analyzed using GraphPad Prism 10 software. For multiple group comparisons, we initially performed one-way ANOVA. When the one-way ANOVA results indicated significant differences between groups ($p < 0.05$), we proceeded with post hoc tests to identify which specific groups exhibited differences. Given that all groups in this study had equal sample sizes ($n = 3$), we selected Tukey's honestly significant difference (HSD) test for multiple comparisons, which is particularly suitable for such cases. This method is widely employed to conduct pairwise comparisons of all possible group mean pairs following a significant ANOVA result, while strictly controlling the family-wise error rate. For pre-planned specific pairwise comparisons, we utilized unpaired t-tests with Bonferroni correction to adjust the significance level (α), accounting for the increased risk of false positives due to multiple comparisons. Specifically, the original significance level ($\alpha = 0.05$) was divided by the number of comparisons performed to obtain the corrected significance threshold. In this study, the significance levels for all statistical tests were established as follows: a corrected $p < 0.05$ was deemed statistically significant (*), $p < 0.01$ as highly significant (**), $p > 0.05$ as non-significant (ns). All figures in the text are labeled with corrected p* values.

References

1. An T, Dugarjaviin M, Han H. Expression and Analysis of TBX3 Gene in the Skin from Three Locations on Dun Mongolian Bider Horse. Genes (Basel). 2024 Dec 11;15(12):1589. doi: 10.3390/genes15121589. PMID: 39766856; PMCID: PMC11675668.
2. Imsland F, McGowan K, Rubin CJ, Henegar C, Sundström E, Berglund J, Schwochow D, Gustafson U, Imsland P, Lindblad-Toh K, Lindgren G, Mikko S, Millon L, Wade C, Schubert M, Orlando L, Penedo MC, Barsh GS, Andersson L. Regulatory mutations in TBX3 disrupt asymmetric hair pigmentation that underlies Dun camouflage color in horses. Nat Genet. 2016 Feb;48(2):152-8. doi: 10.1038/ng.3475. Epub 2015 Dec 21. PMID: 26691985; PMCID: PMC4731265.
