# Supplementary material for: Differential Expression of MITF, WNT3A, SLC7A11, and EDN3 in the Shoulder ‘Bider Marking’ of Dun Mongolian Horses
Source: Animals (Basel). 2026 Mar 19;16(6):967. doi: 10.3390/ani16060967 (PMC13023297; doi:10.3390/ani16060967)
Supplement: Supplementary file 1 [file animals-16-00967-s001.zip › Supplementary Materials Results.pdf]

SI Result

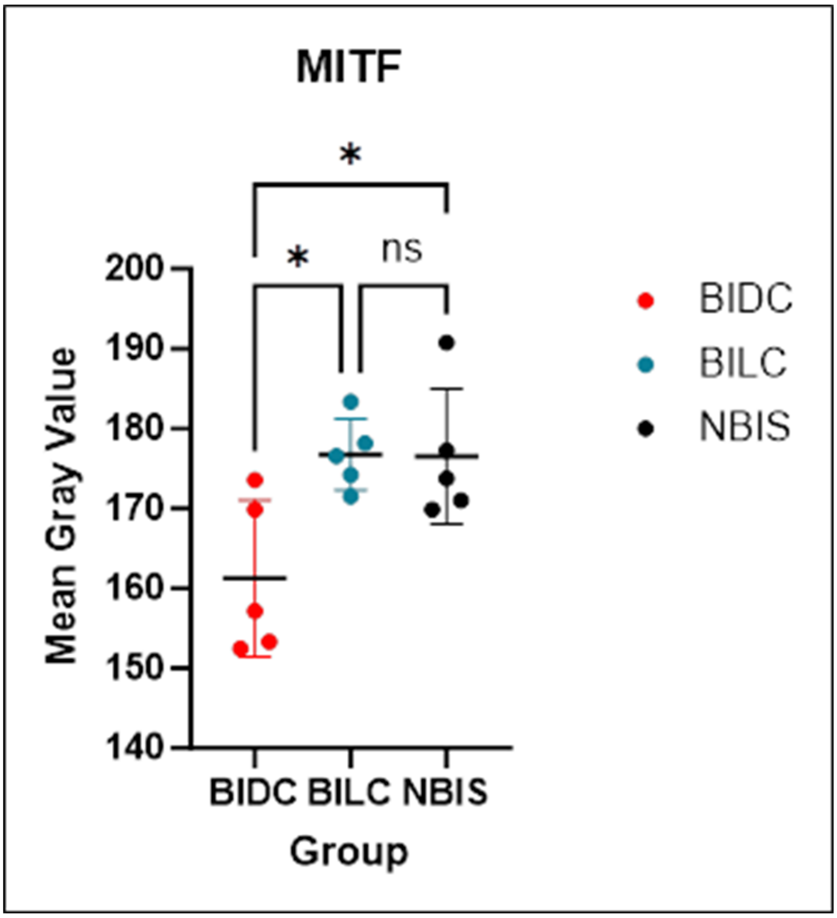

Figure S1. Quantitative analysis of immunohistochemical staining intensity (MITF) in hair bulb. Note: The staining intensity, measured as the mean gray value, was significantly different among the BIDC, BILC, and NBIS groups. Data are presented as the mean ± SD (n=5 randomly selected fields per group). Statistical significance was determined by one-way ANOVA followed by Tukey's post hoc test. (*) $p < 0.05$, (ns) $p > 0.05$.

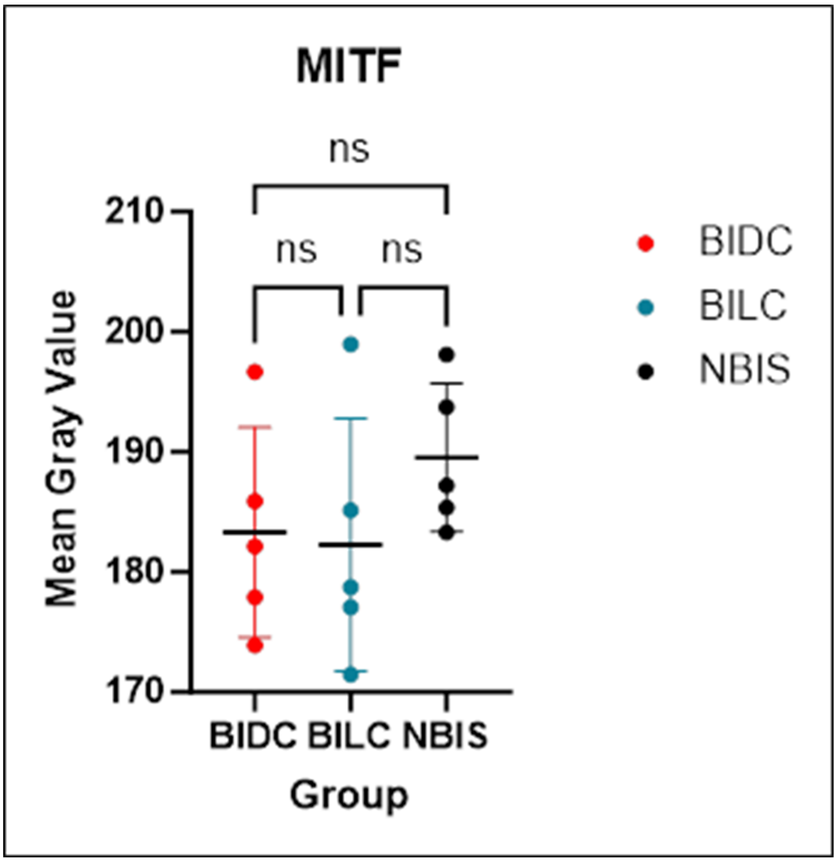

Figure S2. Quantitative analysis of immunohistochemical staining intensity (MITF) in epidermis. Note: The staining intensity, measured as the mean gray value, was significantly different among the BIDC, BILC, and NBIS groups. Data are presented as the mean ± SD (n=5 randomly selected fields per group). Statistical significance was determined by one-way ANOVA followed by Tukey's post hoc test. (ns) p > 0.05.

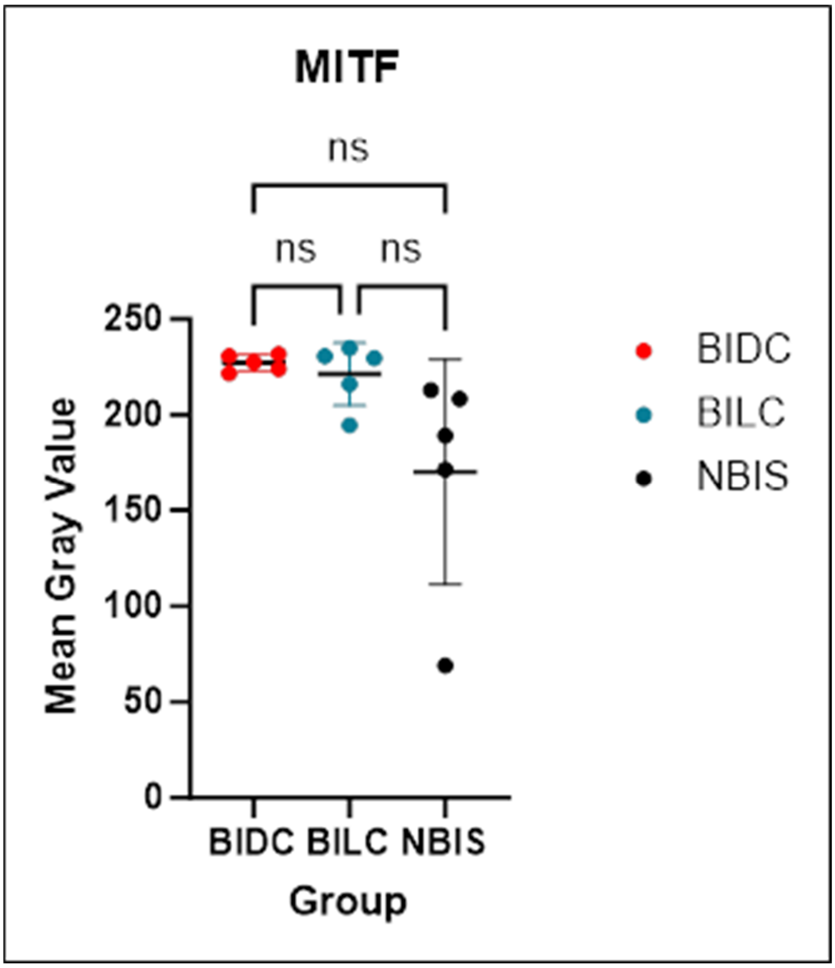

Figure S3. Quantitative analysis of immunofluorescence staining intensity (MITF) in hair bulb. Note: The staining intensity, measured as the mean gray value, was significantly different among the BIDC, BILC, and NBIS groups. Data are presented as the mean ± SD (n=5 randomly selected fields per group). Statistical significance was determined by one-way ANOVA followed by Tukey's post hoc test. (ns) $p > 0.05$.

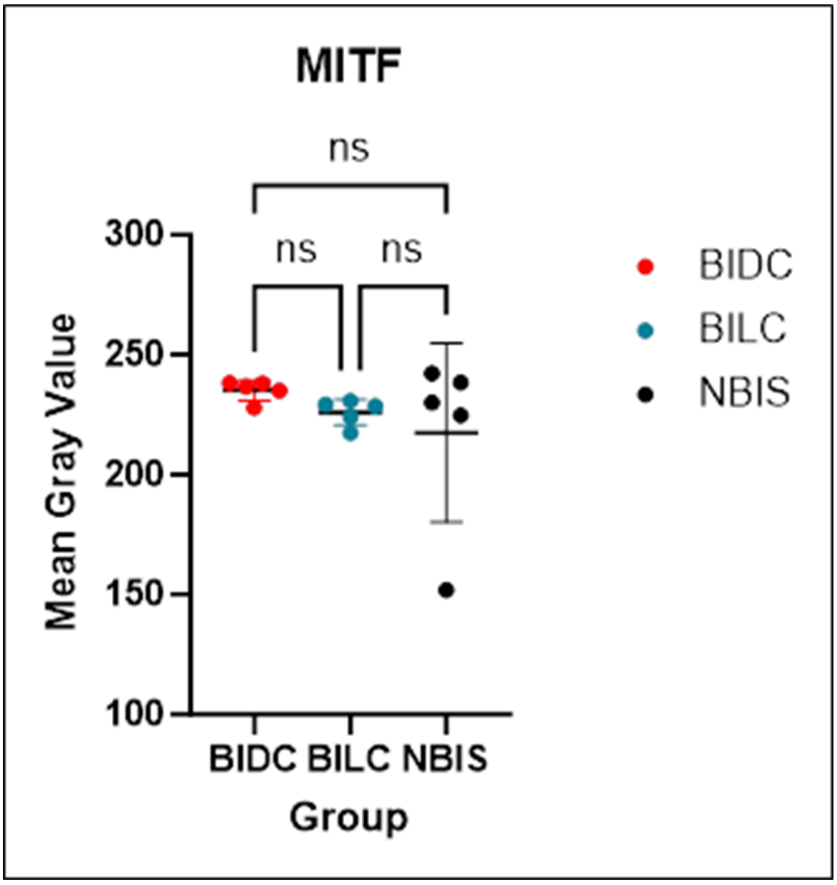

Figure S4. Quantitative analysis of immunofluorescence staining intensity (MITF) in epidermis. Note: The staining intensity, measured as the mean gray value, was significantly different among the BIDC, BILC, and NBIS groups. Data are presented as the mean ± SD (n=5 randomly selected fields per group). Statistical significance was determined by one-way ANOVA followed by Tukey's post hoc test. (ns) $p > 0.05$.

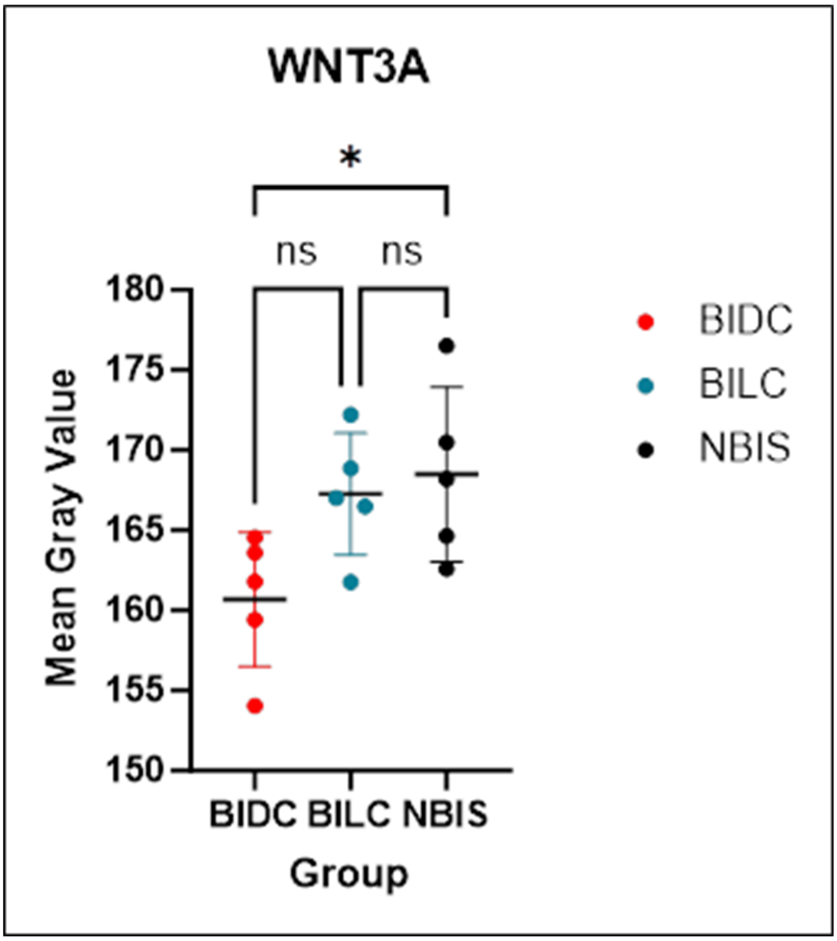

Figure S5. Quantitative analysis of immunohistochemical staining intensity (WNT3A) in hair bulb.

Note: The staining intensity, measured as the mean gray value, was significantly different among the BIDC, BILC, and NBIS groups. Data are presented as the mean ± SD (n=5 randomly selected fields per group). Statistical significance was determined by one-way ANOVA followed by Tukey's post hoc test. *$p < 0.05$, (ns) $p > 0.05$.

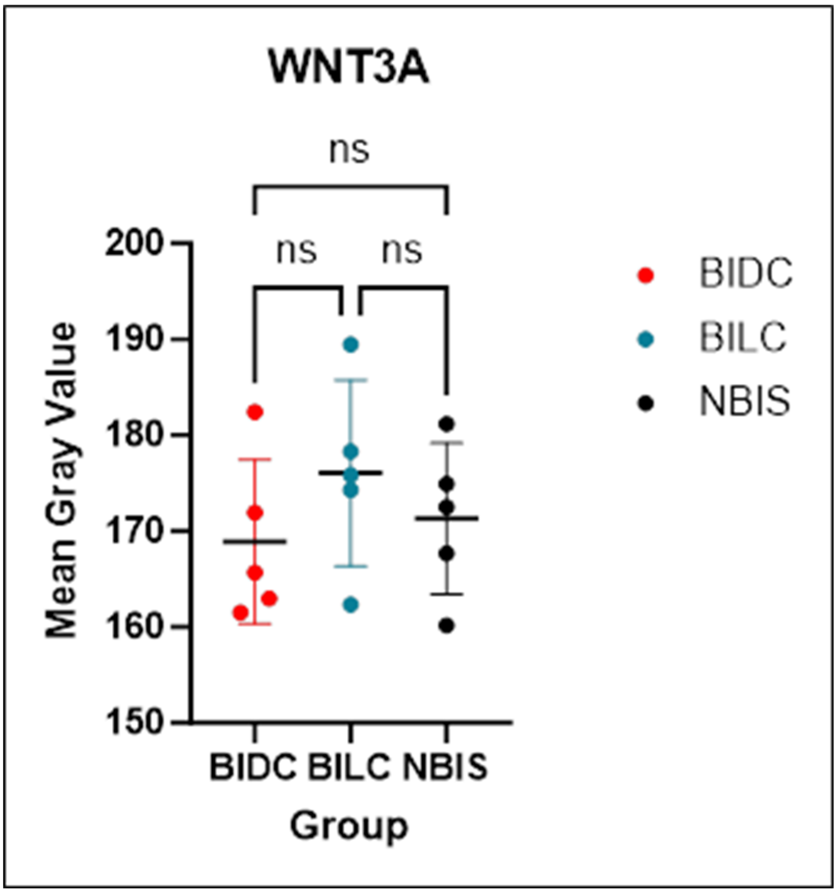

Figure S6. Quantitative analysis of immunohistochemical staining intensity (WNT3A) in epidermis.

Note: The staining intensity, measured as the mean gray value, was significantly different among the BIDC, BILC, and NBIS groups. Data are presented as the mean ± SD (n=5 randomly selected fields per group). Statistical significance was determined by one-way ANOVA followed by Tukey's post hoc test. (ns) $p > 0.05$.

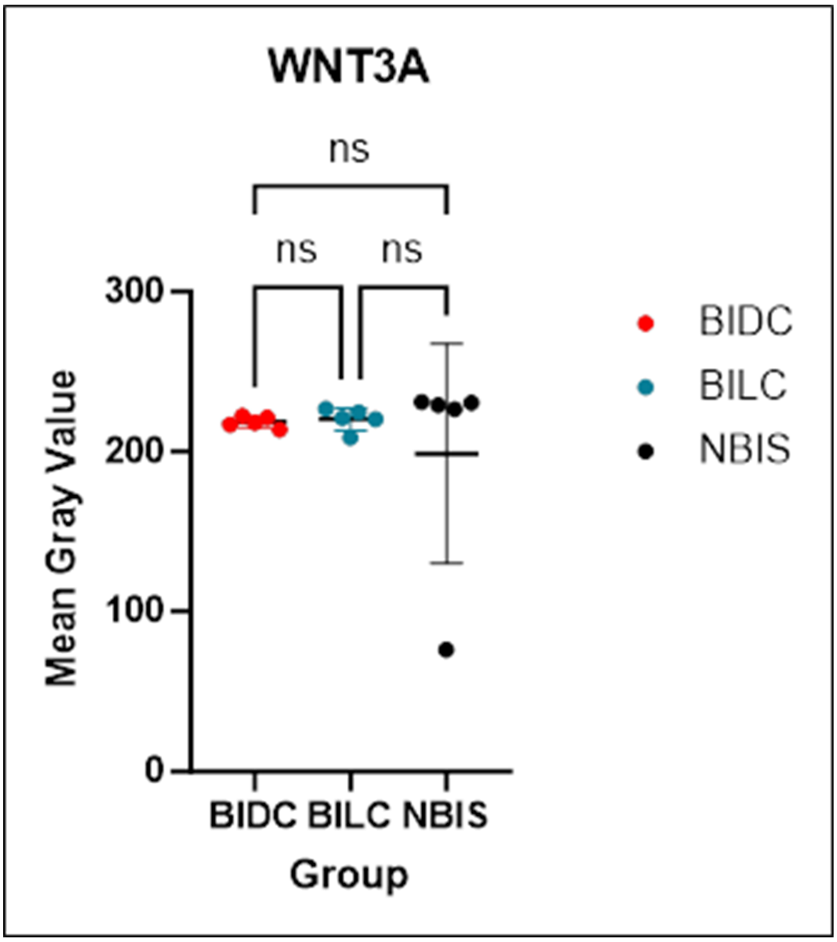

Figure S7. Quantitative analysis of immunofluorescence staining intensity (WNT3A) in hair bulb.

Note: The staining intensity, measured as the mean gray value, was significantly different among the BIDC, BILC, and NBIS groups. Data are presented as the mean ± SD (n=5 randomly selected fields per group). Statistical significance was determined by one-way ANOVA followed by Tukey's post hoc test. (ns) $p > 0.05$.

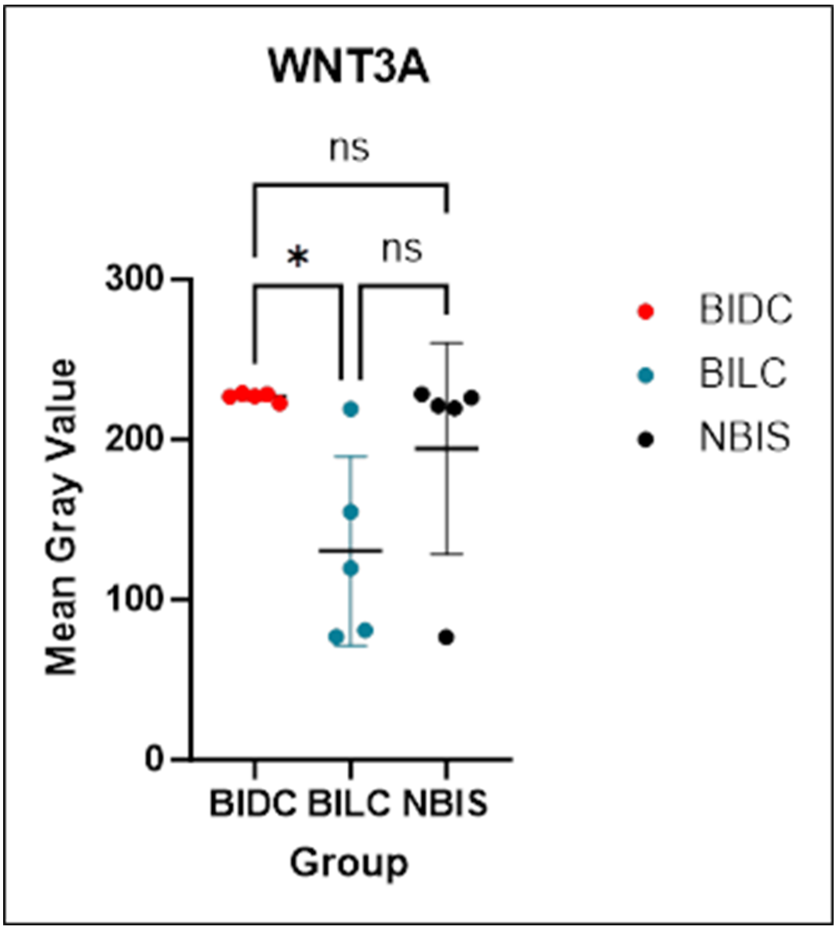

Figure S8. Quantitative analysis of immunofluorescence staining intensity (WNT3A) in epidermis.

Note: The staining intensity, measured as the mean gray value, was significantly different among the BIDC, BILC, and NBIS groups. Data are presented as the mean ± SD (n=5 randomly selected fields per group). Statistical significance was determined by one-way ANOVA followed by Tukey's post hoc test. *$p < 0.05$, (ns) $p > 0.05$.

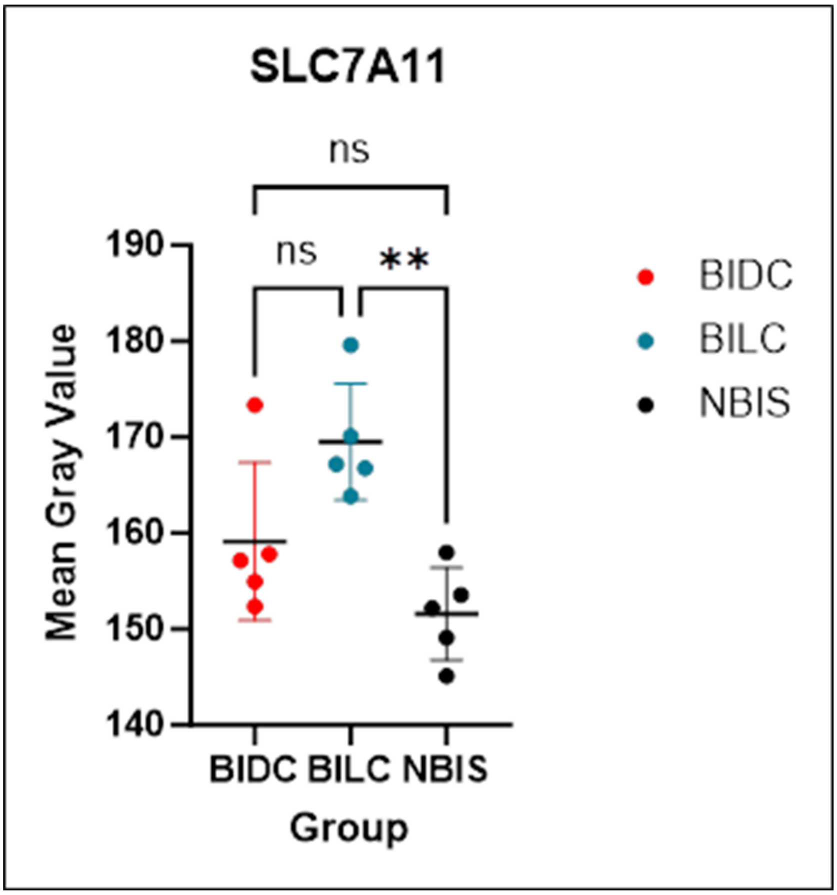

Figure S9. Quantitative analysis of immunohistochemical staining intensity (SLC7A11) in hair bulb.

Note: The staining intensity, measured as the mean gray value, was significantly different among the BIDC, BILC, and NBIS groups. Data are presented as the mean ± SD (n=5 randomly selected fields per group). Statistical significance was determined by one-way ANOVA followed by Tukey's post hoc test. **$p < 0.01$, (ns) $p > 0.05$.

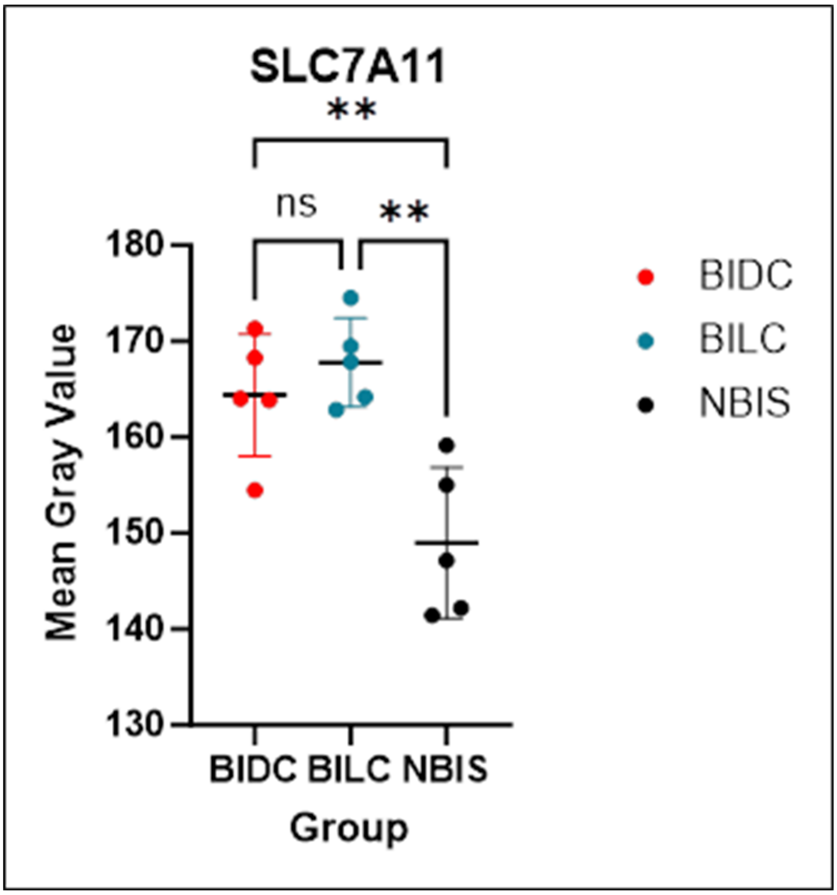

Figure S10. Quantitative analysis of immunohistochemical staining intensity (SLC7A11) in epidermis.

Note: The staining intensity, measured as the mean gray value, was significantly different among the BIDC, BILC, and NBIS groups. Data are presented as the mean ± SD (n=5 randomly selected fields per group). Statistical significance was determined by one-way ANOVA followed by Tukey's post hoc test. $^{**}p < 0.01$, (ns) $p > 0.05$.

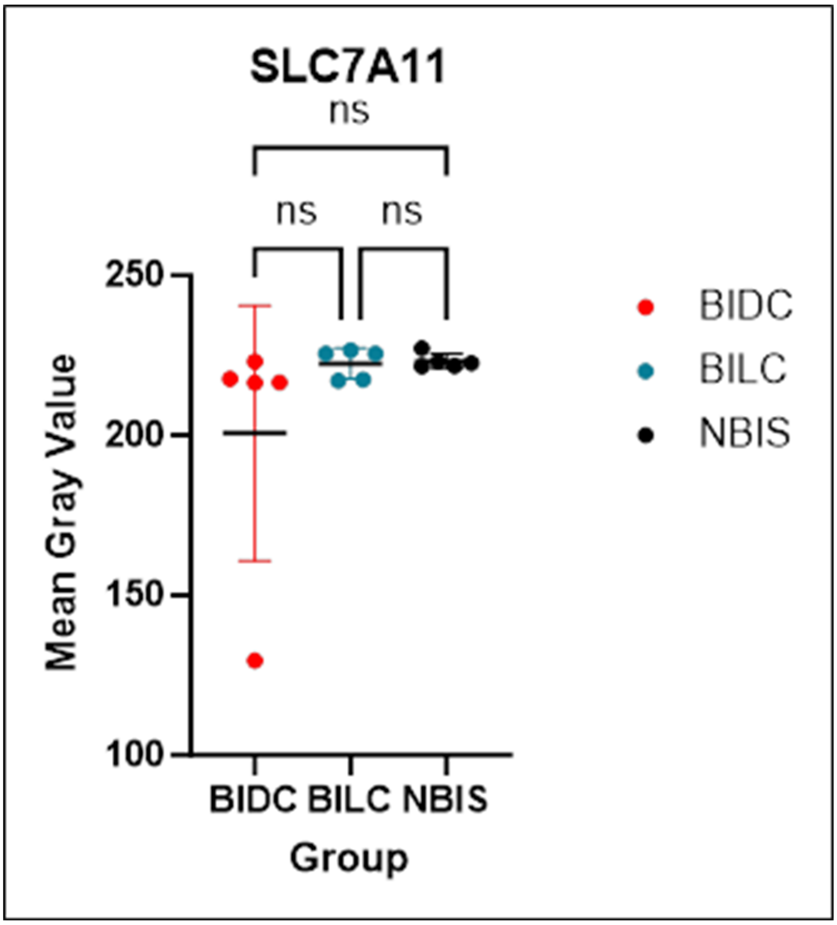

Figure S11. Quantitative analysis of immunofluorescence staining intensity (SLC7A11) in hair bulb.

Note: The staining intensity, measured as the mean gray value, was significantly different among the BIDC, BILC, and NBIS groups. Data are presented as the mean ± SD (n=5 randomly selected fields per group). Statistical significance was determined by one-way ANOVA followed by Tukey's post hoc test. (ns) $p > 0.05$.

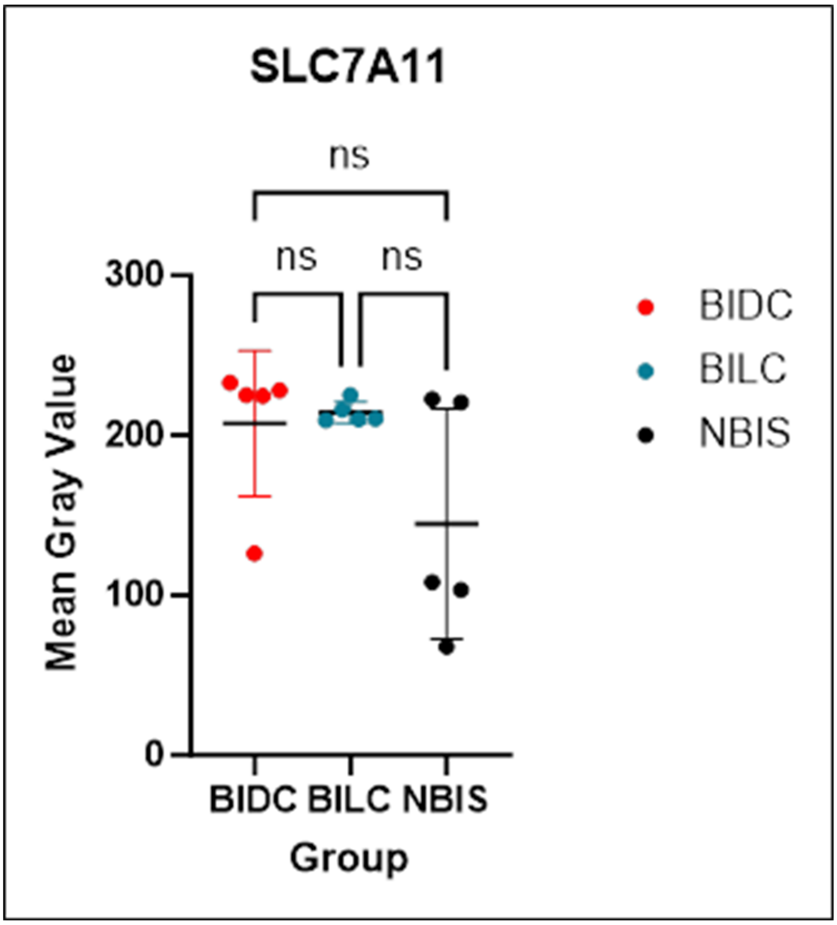

Figure S12. Quantitative analysis of immunofluorescence staining intensity (SLC7A11) in epidermis.

Note: The staining intensity, measured as the mean gray value, was significantly different among the BIDC, BILC, and NBIS groups. Data are presented as the mean ± SD (n=5 randomly selected fields per group). Statistical significance was determined by one-way ANOVA followed by Tukey's post hoc test. (ns) $p > 0.05$.

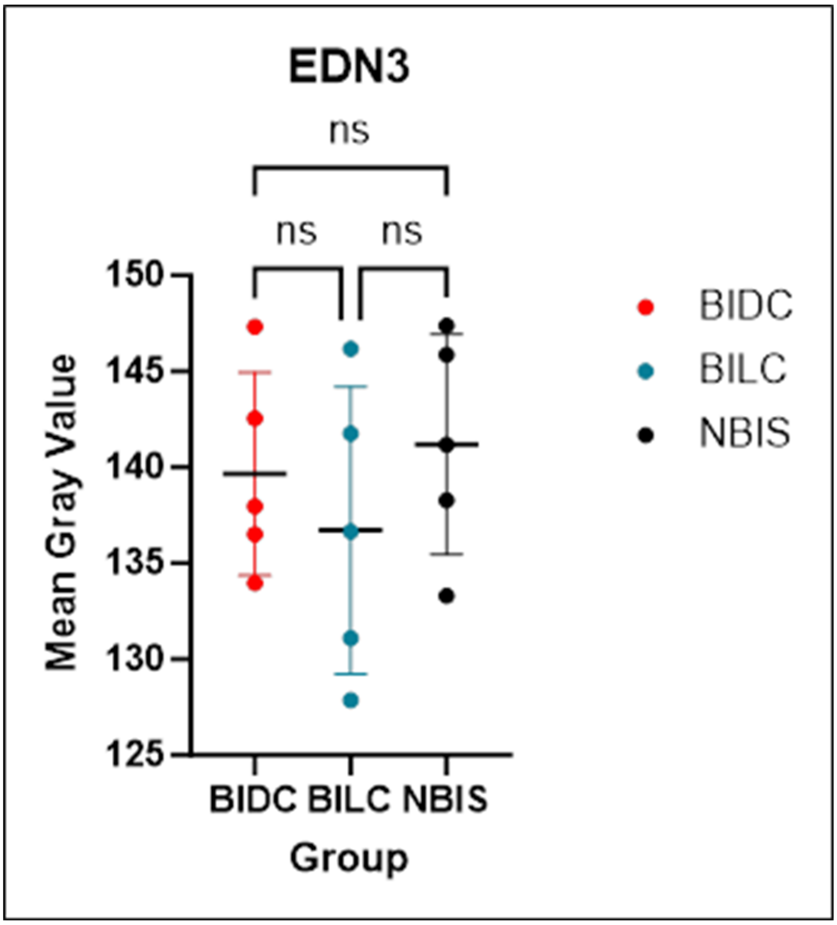

Figure S13. Quantitative analysis of immunohistochemical staining intensity (EDN3) in hair bulb.

Note: The staining intensity, measured as the mean gray value, was significantly different among the BIDC, BILC, and NBIS groups. Data are presented as the mean ± SD (n=5 randomly selected fields per group). Statistical significance was determined by one-way ANOVA followed by Tukey's post hoc test. (ns) $p > 0.05$.

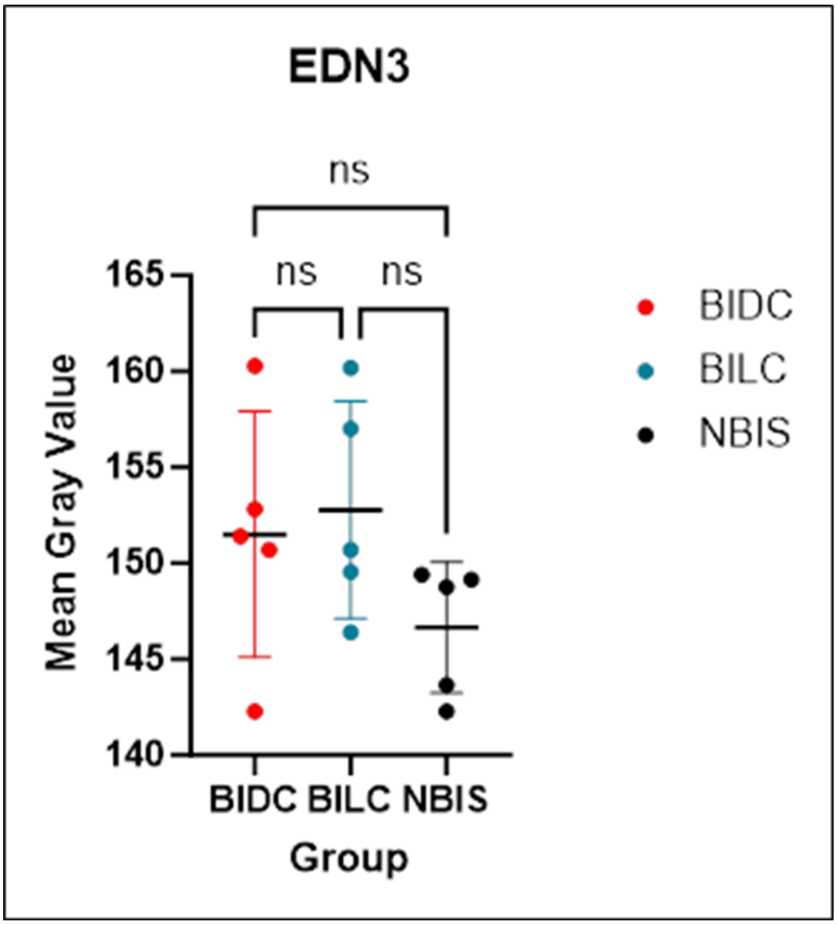

Figure S14. Quantitative analysis of immunohistochemical staining intensity (EDN3) in epidermis.

Note: The staining intensity, measured as the mean gray value, was significantly different among the BIDC, BILC, and NBIS groups. Data are presented as the mean ± SD (n=5 randomly selected fields per group). Statistical significance was determined by one-way ANOVA followed by Tukey's post hoc test. (ns) $p > 0.05$.

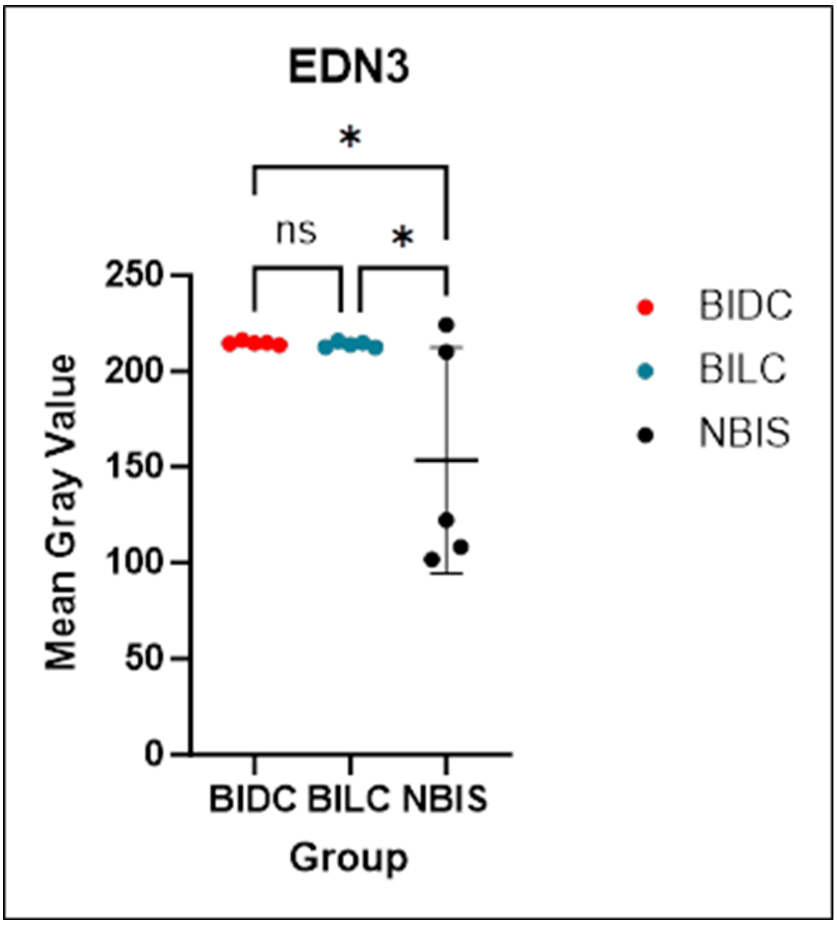

Figure S15. Quantitative analysis of immunofluorescence staining intensity (EDN3) in hair bulb. Note: The staining intensity, measured as the mean gray value, was significantly different among the BIDC, BILC, and NBIS groups. Data are presented as the mean ± SD (n=5 randomly selected fields per group). Statistical significance was determined by one-way ANOVA followed by Tukey's post hoc test. *$p < 0.05$, (ns) $p > 0.05$.

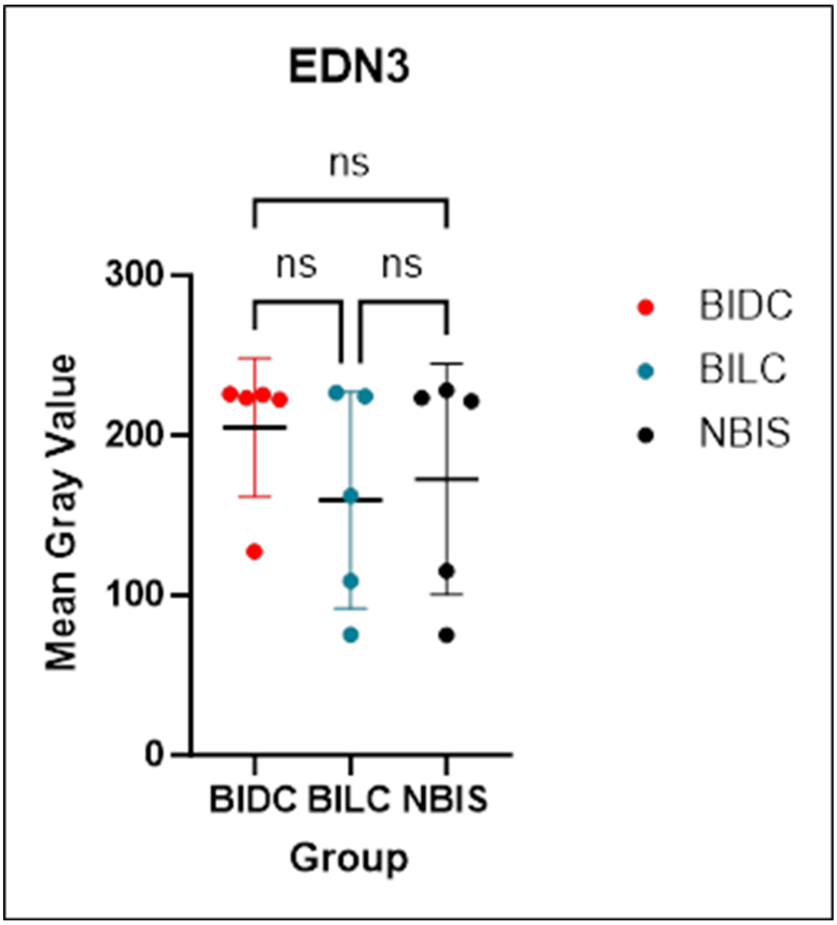

Figure S16. Quantitative analysis of immunofluorescence staining intensity (EDN3) in epidermis.

Note: The staining intensity, measured as the mean gray value, was significantly different among the BIDC, BILC, and NBIS groups. Data are presented as the mean ± SD (n=5 randomly selected fields per group). Statistical significance was determined by one-way ANOVA followed by Tukey's post hoc test. *p < 0.05, **p < 0.01.
